# PIC-GAN: A Parallel Imaging Coupled Generative Adversarial Network for Accelerated Multi-Channel MRI Reconstruction

**DOI:** 10.3390/diagnostics11010061

**Published:** 2021-01-02

**Authors:** Jun Lv, Chengyan Wang, Guang Yang

**Affiliations:** 1 School of Computer and Control Engineering, Yantai University, Yantai 264005, China; ljdream0710@pku.edu.cn; 2 Human Phenome Institute, Fudan University, Shanghai 201203, China; 3 Cardiovascular Research Centre, Royal Brompton Hospital, London SW3 6NP, UK; 4 National Heart and Lung Institute, Imperial College London, London SW7 2AZ, UK

**Keywords:** MRI reconstruction, parallel imaging, generative adversarial network, multi-channel

## Abstract

In this study, we proposed a model combing parallel imaging (PI) with generative adversarial network (GAN) architecture (PIC-GAN) for accelerated multi-channel magnetic resonance imaging (MRI) reconstruction. This model integrated data fidelity and regularization terms into the generator to benefit from multi-coils information and provide an “end-to-end” reconstruction. Besides, to better preserve image details during reconstruction, we combined the adversarial loss with pixel-wise loss in both image and frequency domains. The proposed PIC-GAN framework was evaluated on abdominal and knee MRI images using 2, 4 and 6-fold accelerations with different undersampling patterns. The performance of the PIC-GAN was compared to the sparsity-based parallel imaging (L1-ESPIRiT), the variational network (VN), and conventional GAN with single-channel images as input (zero-filled (ZF)-GAN). Experimental results show that our PIC-GAN can effectively reconstruct multi-channel MR images at a low noise level and improved structure similarity of the reconstructed images. PIC-GAN has yielded the lowest Normalized Mean Square Error (in $\times 10^{-5}$) (PIC-GAN: 0.58 ± 0.37, ZF-GAN: 1.93 ± 1.41, VN: 1.87 ± 1.28, L1-ESPIRiT: 2.49 ± 1.04 for abdominal MRI data and PIC-GAN: 0.80 ± 0.26, ZF-GAN: 0.93 ± 0.29, VN:1.18 ± 0.31, L1-ESPIRiT: 1.28 ± 0.24 for knee MRI data) and the highest Peak Signal to Noise Ratio (PIC-GAN: 34.43 ± 1.92, ZF-GAN: 31.45 ± 4.0, VN: 29.26 ± 2.98, L1-ESPIRiT: 25.40 ± 1.88 for abdominal MRI data and PIC-GAN: 34.10 ± 1.09, ZF-GAN: 31.47 ± 1.05, VN: 30.01 ± 1.01, L1-ESPIRiT: 28.01 ± 0.98 for knee MRI data) compared to ZF-GAN, VN and L1-ESPIRiT with an under-sampling factor of 6. The proposed PIC-GAN framework has shown superior reconstruction performance in terms of reducing aliasing artifacts and restoring tissue structures as compared to other conventional and state-of-the-art reconstruction methods.

## 1. Introduction

Magnetic resonance imaging (MRI) is an important non-invasive imaging modality for in vivo clinical studies that offers preeminent soft tissue contrast without ionizing radiation. However, MRI suffers from long scanning time, especially for high-resolution 3D/4D imaging sequences, which can cause patient discomfort and consequent patient fatigue can yield motion artifacts and thereby degrades the quality of the reconstructed images. Accelerated acquisition and reconstruction are crucial to improve the performance of the current MR imaging techniques. The *k*-space undersampling is a widely used approach to reduce scan time, but it will produce aliasing artifacts in the image domain if reconstructed in a normal way. Hence, various approaches have been explored to obtain accurate reconstructions without introducing aliasing artifacts, including parallel imaging (PI) and compressed sensing (CS).

PI [1] takes use of multi-channel *k*-space data for accelerated imaging. PI techniques can be divided into two categories: (1) reconstruction methods that are performed in the image domain that perform unfolding or reversing [2], and (2) methods that are performed in the *k*-space domain, which require the estimation of missing harmonic data before reconstruction [3]. Since fewer data are acquired in PI, the signal-to-noise ratio (SNR) will be reduced. The SNR of the reconstructed image is related to both the acceleration factor (AF) and the geometry factor (g-factor) [4]. It is well known that the g-factor [5] depends on the geometrical distribution of the receiver coils as well as the sampling patterns [6]. In contrast, CS algorithms adopt a nonlinear process to reconstruct images from undersampled *k*-space data. It is assumed that the signal is sparse [7,8] in a particular transform domain, e.g., via wavelet [9] or total variation [10,11,12,13], and the artifacts generated by the random sampling are incoherent [7]. Although PI and CS based methods can shorten the acquisition time, both of them require long reconstruction time due to the iterative computations.

Recent studies have demonstrated that deep learning-based MRI reconstruction algorithms are capable to recover high-quality images from undersampled acquisitions with significantly reduced reconstruction time. Wang et al. [14] trained a convolutional neural network (CNN) architecture to identify the mapping between zero-filled (ZF) images and fully-sampled images. Sun et al. [15] presented an ADMM-NET that learned parameters in the alternating direction method of multipliers (ADMM) algorithm via a back-propagation. Schlemper et al. [16] introduced a deep cascade of CNNs that intercalated data consistency layers for dynamic 2D cardiac MRI reconstruction with Cartesian undersampling. Instead of learning the artifact-free images, Lee et al. [17] combined CNN with PI to estimate the image degrading patterns and then removed the corresponding artifacts. Furthermore, Lv et al. [18] developed a stack of autoencoders to remove streaking artifacts from radial undersampled free-breathing 3D abdominal MRI data. More recent studies [19,20] integrated the attention mechanism into CNN for accelerated MRI reconstruction, which improved the reconstruction outcome by taking advantage of long-range dependencies across images.

Nowadays, generative adversarial network (GAN) based models have been exploited to perform MRI reconstruction. GAN consists of a generator and a discriminator. The generator is trained to learn the distribution from the giving dataset, while the discriminator is trained to distinguish the generated images from the real ones. Since the error of the discriminator is backpropagated to the generator, the error of the discriminator and the generator conflicts, resulting in an adversarial loss. Compared to other loss functions, the use of adversarial loss can improve the perceptual image quality. Shitrit et al. [21] presented a GAN-based model to reconstruct MR images directly from under-sampled *k*-space data. The generator is capable of estimating the missing *k*-space data and the discriminator is used to judge the generated samples from the real ones. Yang et al. [22] proposed a deep de-aliasing generative adversarial network named DAGAN, which adopted a residual U-Net as generator with a loss function consists of an image domain loss, a frequency domain loss, a perceptual loss and an adversarial loss. Quan et al. [23] proposed a GAN-based framework with a cyclic loss. This framework was composed of two consecutive networks, one was used to reconstruct the under-sampled *k*-space data and the other was used to refine the result. Jiang et al. [24] proposed a de-aliasing fine-tuning Wasserstein generative adversarial network (DA-FWGAN) to perform CS-MRI reconstruction. This approach combines fine-tuning and Wasserstein distance for training. In addition, Cole et al. [25] proposed an unsupervised GAN framework for MRI reconstruction that does not rely on fully-sampled datasets for supervision. Yuan et al. [26] developed a self-attention GAN that combines the Self-Attention mechanism with Relative Average discriminator (SARA-GAN) for under-sampled k-space data reconstruction. Thanks to the long-range global dependence constructed by the self-attention module, this approach can reconstruct images with more realistic image details and higher quantitative metrics.

To the best of our knowledge, most previous approaches have used single-channel data for training. In fact, multi-channel technology provides many complementary information. Several endeavors have been made to extend the previous single-channel CNN-based MRI reconstruction methods to the multi-channel reconstructions. Hammernik et al. [27] presented a variational network (VN) for multi-channel MRI reconstruction. Subsequently, Zhou et al. [28] developed a PI-CNN reconstruction framework, which utilized a cascaded structure that intercalated the CNN and PI-DC layers. This method allows the network to make better use of information from multi-coils. Nevertheless, the multi-channel loss function was not integrated into the architecture of the network. Wang et al. [29] trained a deep complex CNN that yielded the direct mapping between aliased multi-channel images and fully-sampled multi-channel images. Unlike other networks for PI, no prior information (such as sparse transform or coil sensitivity) was required, and therefore could provide an end-to-end network in this deep complex CNN based framework. It is of note that all these studies have focused on a single-domain (in either the image domain or the *k*-space domain).

In this study, we aim to introduce a novel reconstruction framework named ’Parallel Imaging Coupled Generative Adversarial Network (PIC-GAN)’, which is developed to learn a unified model for improving multi-channel MRI reconstruction. We performed experiments on two MRI datasets (abdominal and knee MRI datasets) to validate the efficacy and generalization capacity of the proposed method with different acceleration factors and different sampling trajectories. Besides, we compared our model with the conventional sparsity-based parallel imaging method (L1-ESPIRiT), the VN model and the GAN approach with single-channel images as input (ZF-GAN).

## 2. Methods

### 2.1. Problem Formulation

The idea of PI is to apply coil sensitivity encoding into the reconstruction of multi-channel undersampled *k*-space data. The PI reconstruction can be formulated as an inverse problem, which can be described in a matrix-vector form:

(1) y=Ex+n=RℑSx+n,

where *y* represents the *k*-space measurements, *x* represents the image to be reconstructed, n represents the noise, E represents the forward encoding operator including the sampling trajectory R, the Fourier transform *ℑ*, and the coil sensitivity S.

The presence of the operator E and n causes the solution of Equation (1) to be ill-posed [30]. Thus, Equation (1) is usually solved in an iterative manner with the inclusion of certain regularization terms:

(2) $$\min_{x}\frac{1}{2}{∥Ex-y∥}_{2}^{2}+\sum_{i}\lambda_{i}R_{i}\left(x\right),$$

where ${∥\cdot ∥}_{2}^{2}$ denotes the $l_{2}$ norm, $R_{i}$ represents the *i*-th regularization term and $\lambda_{i}$ represents the corresponding weighting parameter. The regularization term $R_{i}$ is typically selected as a $l_{1}$-norm in CS reconstruction [31,32,33]. ADMM [15] algorithm is usually employed to solve this optimization problem.

Recently, with the introduction of deep learning, $R_{i}$ can be formulated as a CNN based regularization term, where the model parameters can be trained from existing dataset.

(3) $$\min_{x}\frac{1}{2}{∥Ex-y∥}_{2}^{2}+\lambda {∥x-F_{\mathrm{CNN}}\left(x_{u};\theta \right)∥}_{N}.$$

Here, $x_{u}$ represents an undersampled image to be reconstructed, $F_{\mathrm{CNN}}\left(x_{u};\theta \right)$ is an image generated by the CNN network and θ represents the optimal parameters of the trained CNN.

Our objective is to train a generator G that can generate a fully-reconstructed MR image ${\hat{x}}_{u}=G_{\theta_{G}}\left(x_{u}\right)$ from a zero-filled reconstruction image $x_{u}$ under the constraint that $G_{\theta_{G}}\left(x_{u}\right)$ is indistinguishable from the image reconstructed from the fully-sampled *k*-space data ($\hat{x}$).

The objective function of D is to maximize the log-likelihood for estimating the conditional probability, where $D\left(G\left(x_{u}\right)\right)=0$ and $D(\hat{x})=1$. Hence, this can be addressed by defining an adversarial loss $L_{adv}$, which can be rewritten as a minimax problem between the generator $G_{\theta_{G}}\left(x\right)$ and $D_{\theta_{D}}\left(x\right)$. The training process of GAN can be parameterized by $\theta_{G}$ and $\theta_{D}$ as following

(4) $$\min_{\theta_{G}}\max_{\theta_{D}}L_{adv}\left(\theta_{D},\theta_{G}\right)=E_{\hat{x}∼P_{\mathrm{train}}\left(\hat{x}\right)}\left[\log D_{\theta_{D}}\left(x\right)\right]+E_{x_{u}∼P_{G}\left(x_{u}\right)}\left[\log\left(1-D_{\theta_{D}}\left(G_{\theta_{G}}\left(x_{u}\right)\right)\right)\right].$$

Here, $x_{u}$ is sampled from a fixed latent distribution $P_{G}\left(x_{u}\right)$ and real samples $\hat{x}$ come from a real data distribution $P_{train}\left(\hat{x}\right)$. Once the training converges, $G_{\theta_{G}}$ can generate the image $G_{\theta_{G}}\left(x_{u}\right)$ which is similar to $\hat{x}$, and $D_{\theta_{D}}$ is unable to differentiate between them.

### 2.2. The Proposed PIC-GAN Reconstruction Framework

**Figure 1:**
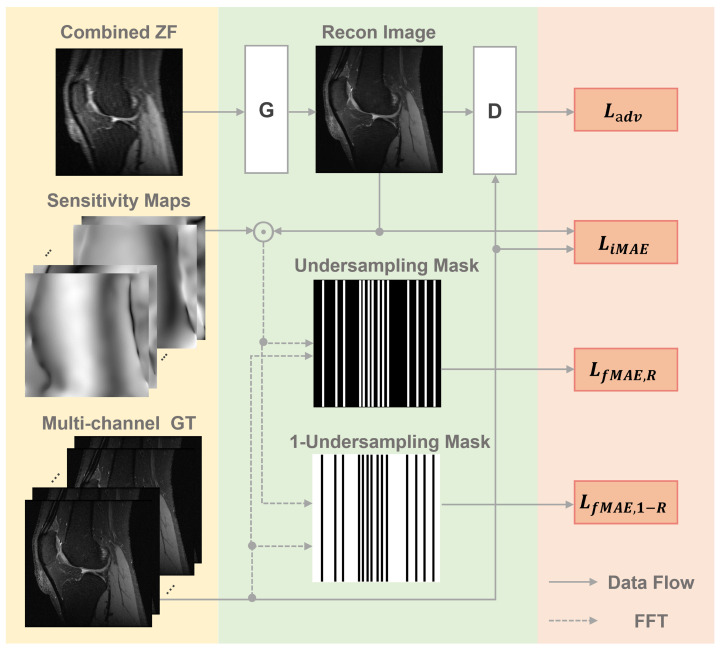
Schema of the proposed parallel imaging and generative adversarial network (PIC-GAN) reconstruction network.

The schema of the proposed PIC-GAN for multi-channel image reconstruction is illustrated in Figure 1. The detailed architecture of G and D components are described as following. The input to the generator is a single, sensitivity-weighted recombined image $x_{u}$. Besides, the input is made up of two channels, the real and the imaginary parts.

**Figure 2:**
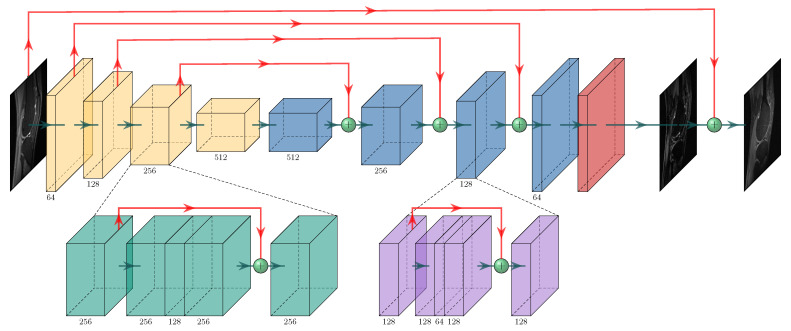
The generator G consists of four encoder blocks followed by corresponding 4 decoder blocks. In addition, shortcut connections are applied to connect mirrored layers between the encoder and decoder paths.

A deep residual U-Net is adopted for the generator to improve learning robustness and accuracy. As shown in Figure 2, the model of Generator G consists of a network of a convolutional encoder and a network of convolutional decoder, and there are multiple shortcut connections between them. The encoder blocks (colored in yellow) are capable to compress the input images and explore the image features with strong robustness and spatial invariance. The decoder blocks (colored in blue) is utilized to restore image features and increase image resolution. Multiple shortcut connections (red lines) are introduced to connect the feature maps from the encoder to the decoder, thus feeding different levels of features to the decoder to get better image reconstruction details. The final result is calculated by adding the zero-filled image $x_{u}$ to the output of generator $G(x_{u})$. More specifically, each encoder block (colored in green) or decoder block (colored in lavender) consists of four convolutional layers with a kernel size of 3×3 and different numbers (illustrated under the blocks) of feature maps. It is then followed by a convolutional layer without any activation to get two output channels for the real and imaginary parts, respectively.

A discriminator is connected to the generator output. The discriminator D network is composed of similar encoding part of the generator G, which consists of 6 convolutional layers. In all the convolutional layers except the last one, each convolutional layer is followed by batch normalization (BN) and ReLU layers. We use 64, 128, 256, 512 feature maps for the first 4 layers. Meanwhile, a convolution with a stride of 2 is used to reduce the image resolution. The first four layers use kernel size of 3×3, while the last layer uses kernel size of 1×1. The final layer simply averages out features of the seventh layer to obtain decision variables for binary classification without soft-max operation. The output of the last residual block is used to calculate the mentioned adversarial loss $L_{adv}$.

In this study, we incorporate parallel imaging into the GAN paradigm to fully utilize all the information acquired from the multi-channel coils. Meanwhile, the data consistency loss is designed for training the generator *G* in both frequency and image domains to help the optimization and to exploit the complementary properties of the two domains. This loss consists of three parts (Figure 1), one is a pixel-wise image domain mean absolute error (MAE) $L_{i\mathrm{MAE}}\left(\theta_{G}\right)$, the other two are frequency domain MAE losses $L_{i\mathrm{MAE},R}\left(\theta_{G}\right)$ and $L_{f\mathrm{MAE},1-R}\left(\theta_{G}\right)$. The three loss functions can be written as

(5) $$L_{i\mathrm{MAE}}\left(\theta_{G}\right)=\sum_{q}{∥x^{q}-S^{q}{\hat{x}}_{u}∥}_{1},$$

(6) $$L_{f\mathrm{MAE},R}\left(\theta_{G}\right)=\sum_{q}{∥y_{R}^{q}-RℑS^{q}{\hat{x}}_{u}∥}_{1},$$

(7) $$L_{f\mathrm{MAE},1-R}\left(\theta_{G}\right)=\sum_{q}{∥y_{1-R}^{q}-\left(1-R\right)ℑS^{q}{\hat{x}}_{u}∥}_{1}.$$

Here, *q* denotes the coil element, the $L_{i\mathrm{MAE}}\left(\theta_{G}\right)$ term removes aliasing artifacts between the reconstructed image and its corresponding ground truth image. Specifically, the $L_{f\mathrm{MAE},R}\left(\theta_{G}\right)$ term guarantees that the reconstructed image produces corresponding undersampled image matching the undersampled *k*-space measurements ($y_{R}$). The $L_{f\mathrm{MAE},1-R}\left(\theta_{G}\right)$ term ensures that the difference between the unacquired *k*-space data ($y_{1}-R$) and interpolated data based on reconstruction to be minimal.

Together with $L_{\mathrm{adv}}$, the complete loss function can be written as:

(8) $$L_{\mathrm{total}\;}=L_{\mathrm{adv}}\left(\theta_{D},\theta_{G}\right)+\alpha L_{\mathrm{iMAE}}\left(\theta_{G}\right)+\beta L_{\mathrm{fMAE},R}\left(\theta_{G}\right)+\gamma L_{\mathrm{fMAE},1-R}\left(\theta_{G}\right).$$

Here, α, β and γ are the hyper-parameters that control the trade-off between each function. The adversarial loss term $L_{\mathrm{adv}}$ enforces the reconstructed images to keep the high perceptual quality and to maintain image details and textural information of the images.

It is well known that the GAN model is hard to be trained [23] due to the need for alternate training process on the adversarial components. Inspired by the study of DAGAN [22], we incorporated the refinement learning to stabilize the training of our model. In fact, we utilize ${\hat{x}}_{u}=G_{\theta_{G}}\left(x_{u}\right)+x_{u}$. Thus, the generator only generates information that is not sampled, which can greatly reduce the complexity of the model.

#### 2.2.1. Datasets

To validate the efficacy and generalization capacity of our proposed method, publicly available abdominal [34] and knee [35] MRI datasets are used retrospectively. Both datasets were acquired from a GE 3.0 T whole-body scanner (GE Healthcare, Milwaukee, WI, USA). Using the same PIC-GAN architecture, we trained our model on each dataset and test independently on their corresponding testing dataset.

The abdominal MRI dataset contains images acquired from 28 subjects. The signal was acquired by a 32-channel pediatric coil. The data was undersampled by a 3D spoiled-gradient-echo with Poisson-disc random undersampling of the phase encodes. The imaging parameters were TE/TR = 1.128 ms/4.832 ms, field-of-view (FOV) = 38 × 38 cm${}^{2}$, slice thickness = 2 mm, flip angle = 15${}^{\circ }$, bandwidth = ±64 kHz, matrix size = 308 × 230 × 156, and auto-calibration signal (ACS) lines = 24 × 20.

The knee dataset consists of images acquired from 20 subjects. The MRI data were acquired with an 8-channel knee coil. The images were fully sampled using a 3D FSE CUBE sequence with proton density weighting. The imaging parameters were TE/TR = 0.944 ms/3.832 ms, FOV = 35 × 35 cm${}^{2}$, slice thickness = 2 mm, flip angle = 15${}^{\circ }$, bandwidth = ±64 kHz, and matrix size = 192 × 224 × 184.

In this study, the real and imaginary components of the complex MR image $x_{u}$ were considered as two individual image channels. Among all the 28 abdominal, 26 subjects were randomly selected for training, and the remaining 2 subjects were used for test. For each subject, 50 central slices were selected. Thus, the training set contained 1300 slices and the test set had 100 slices. Similarly, 18 out of 20 knee data were randomly selected for training, while the remaining subjects were used for test. A total of 100 central slices were selected for each subject. Therefore, the knee training and test sets contained 1800 and 200 images, respectively.

#### 2.2.2. Comparison Studies, Experimental Settings and Evaluation

The proposed PIC-GAN was tested on data with both regular and random Cartesian undersampling under 2×, 4× and 6× acceleration factors. Next, we evaluated the performance of the PIC-GAN against previously proposed reconstruction methods, including L1-ESPIRiT, VN and ZF-GAN. The L1-ESPIRiT reconstruction was performed using the Berkeley Advanced Reconstruction Toolbox (BART) [36], where the parameters were optimized for the best SNR performance. The coil sensitivity maps were estimated by ESPIRiT [37] with 24 and 40 calibration lines for abdominal and knee dataset, respectively.

We trained the networks with the following hyperparameters: α=1 and β=γ=10 for PIC-GAN reconstruction. For the ZF-GAN method, reconstruction was performed without using sensitivity maps. The Adam optimizer [7] is used for the training. The model used a batch size of 32 and the initial learning rate of $10^{-4}$ for training, which decreased monotonically over 2000 epochs. The model with the highest validation Peak Signal to Noise Ratio (PSNR) was selected for testing.

Experiments were carried out on a system equipped with GPUs of NVIDIA Tesla V100 (4 cores, each with 16 GB memory) and a 32-core Intel-Xeon Gold-6130-CPU at 2.10 GHz. Our PIC-GAN was developed using Tensorpack [38] with the Tensorflow [39] library.

We evaluated the reconstruction results quantitatively in terms of Peak Signal to Noise Ratio (PSNR), Normalized Mean Square Error (NMSE), and Structural Similarity Index (SSIM). A paired Wilcoxon signed-rank test was conducted to compare the NMSE, PSNR and SSIM measurements between different approaches. *p* < 0.05 was treated as statistically significant.

## 3. Results

### 3.1. Reconstruction Results: Abdominal MRI Data

**Figure 3:**
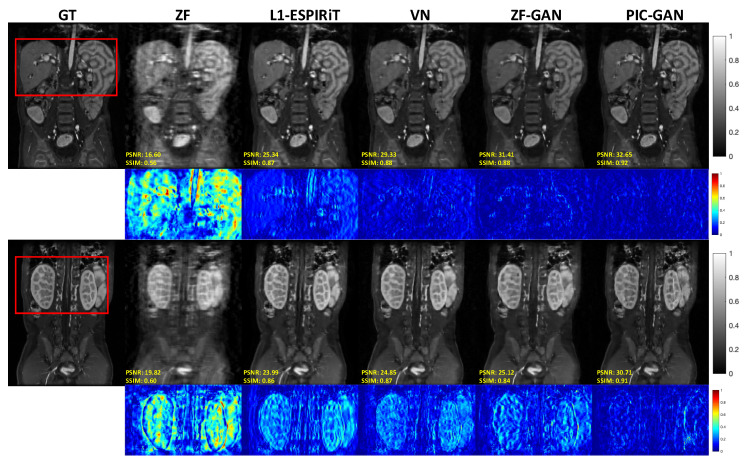
Representative abdominal images reconstructed with acceleration AF = 6. The first and second rows depict reconstruction results for regular Cartesian sampling, the third and fourth row depict the same for variable density random sampling. The PIC-GAN reconstruction shows reduced artifacts compared to other methods. (GT: Ground truth. ZF: Zero-filled. L1-ESPIRiT: Sparsity-based parallel imaging. VN: Variational network. ZF-GAN: Conventional GAN with single-channel images as input PIC-GAN: Our proposed method. Red box: Zoomed-in area.)

Figure 3 shows representative images reconstructed from ZF, L1-ESPIRiT, VN, ZF-GAN, and PIC-GAN with sixfold undersampling compared to the ground truth (GT). As illustrated in the 1st and 3rd rows, the liver and kidney regions are marked with red boxes. The ZF reconstruction was remarkably blurred. Zoomed in error maps showed that liver vessels almost disappeared in L1-ESPIRiT. Moreover, the VN reconstructed images contained substantial residual artifacts, which can be seen in the error maps. The ZF-GAN results produced unnatural blocky patterns for vessels and appeared blurrier at image edges. Compared to the other methods, PIC-GAN results had the least error and were capable of removing the aliasing artifacts. Correspondingly, the proposed PIC-GAN method also performed the best in terms of PSNR and SSIM metrics. These observations have a good correlation with the numerical analysis shown in Table 1.

### 3.2. Reconstruction Results: Knee MRI Data

**Figure 4:**
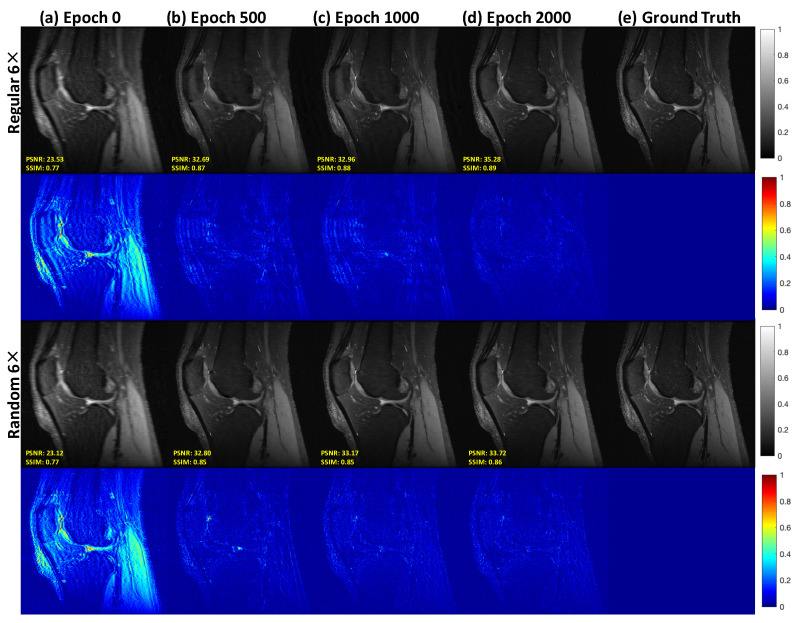
Visualization of the intermediate results of our PIC-GAN reconstruction. (**a**) Undersampled image with an acceleration factor of 6× with the regular (1st row) and the random (3rd row) Cartesian sampling (**b**–**d**) Results from intermediate steps 500 to 2000 in the reconstruction process. (**e**) Ground truth.

To better understand the refining procedure of our PIC-GAN, the intermediate results during the iterations of the reconstruction are shown in Figure 4. We can observe a gradual improvement in the quality of the reconstruction from epochs 0 to 2000, which is consistent with the quantitative results (PSNR and SSIM) showing in the sub-figures in Figure 4.

**Figure 5:**
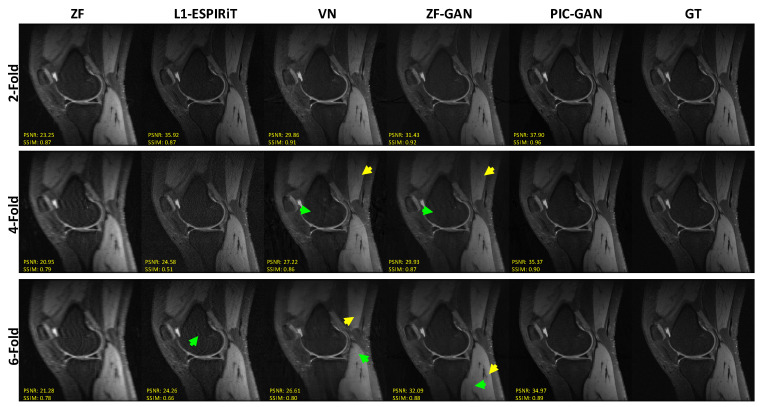
Comparison of different reconstruction methods with different acceleration factors for the knee dataset. From left to right, each column represents selected knee image reconstructed using ZF, L1-ESPIRiT, VN, ZF-GA and PIC-GAN, respectively, compared to the GT. (GT: Ground truth. ZF: Zero-filled. L1-ESPIRiT: Sparsity-based parallel imaging. VN: Variational network. ZF-GAN: Conventional GAN with single-channel images as input PIC-GAN: Our proposed method. The ZF-GAN reconstructed images were over-smoothed with blocky artifacts (yellow arrows) and obvious residual artifacts (green arrows).)

Figure 5 shows representative images reconstructed from ZF, L1-ESPIRiT, VN, ZF-GAN and the proposed PIC-GAN compared to the GT. All four methods (L1-ESPIRiT, VN, ZF-GAN and the proposed PIC-GAN) achieved acceptable image quality when AF was selected as 2. When 4-fold undersampling was applied, the residual artifacts can be clearly observed in images reconstructed using VN. Besides, the images reconstructed by ZF-GAN appeared less noisy than L1-ESPIRiT and VN. However, the ZF-GAN reconstructed images were over-smoothed with blocky artifacts (yellow arrows) and obvious residual artifacts (green arrows) as shown in Figure 5. The proposed PIC-GAN, on the other hand, could better maintain fine details and thus show more accurate textures. The proposed PIC-GAN method achieved the highest PSNR with acceleration of factor up to 6. The other two methods missed some high-frequency texture details (green and yellow arrows). Compared to other reconstruction approaches, PIC-GAN yielded the lowest NMSE and the highest PSNR with regular under-sampling.

**Figure 6:**
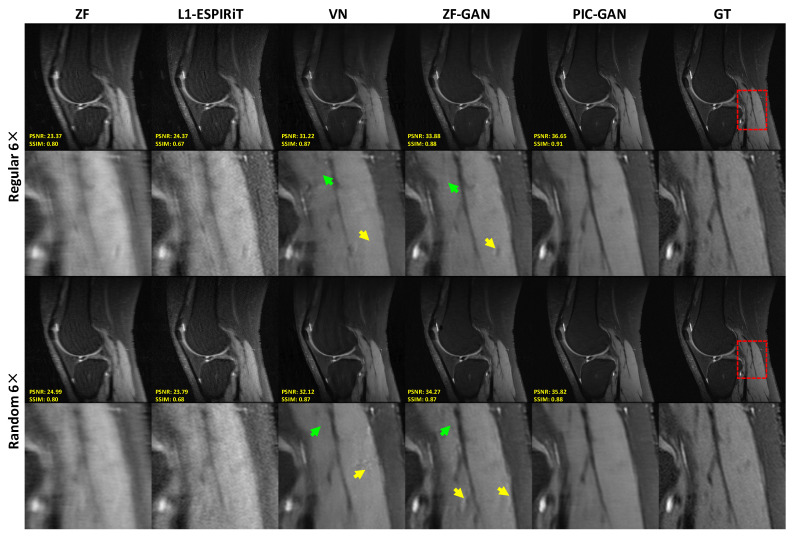
Representative knee images reconstructed with an acceleration factor of 6. The first and second rows show reconstruction results using regular Cartesian sampling, the third and fourth rows show reconstruction results using variable density random sampling. Zoomed in views (as red boxes) show that the proposed method has resulted in both sharper and cleaner reconstruction compared to the results obtained by L1-ESPIRiT, VN and ZF-GAN. Both ZF-GAN and PIC-GAN reconstruction can significantly suppress the artifacts compared to ZF and L1-ESPIRiT. (GT: Ground truth. ZF: Zero-filled. L1-ESPIRiT: Sparsity-based parallel imaging. VN: Variational network. ZF-GAN: Conventional GAN with single-channel images as input PIC-GAN: Our proposed method. ZF-GAN images contained blurred vessels (green arrows) and blocky patterns (yellow arrows).)

Figure 6 demonstrates the advantage of the proposed PIC-GAN method using different sampling patterns. The ZF reconstructed images presented with a significant amount of aliasing artifacts. Similarly, there were significant residual artifacts and amplified noise that existed in the results obtained by L1-ESPIRiT. For the reconstruction produced by VN, fine texture details were missing, which might limit the clinical usage. The ZF-GAN images enhanced the spatial homogeneity and the sharpness of the images reconstructed by VN. However, ZF-GAN images contained blurred vessels (green arrows) and blocky patterns (yellow arrows). The PIC-GAN not only suppressed aliasing artifacts but also provided sharper edges and more realistic texture details. These observations are consistent with the quantitative analyzed results shown in Table 2.

### 3.3. Quantitative Evaluations

Table 1 and Table 2 show the quantitative metrics, including PSNR, SSIM, NMSE, and the reconstruction time, for all compared methods. The numbers in Table 1 and Table 2 represent the mean values and standard deviation of corresponding metrics (bold numbers indicate the best performance). Compared to the L1-ESPIRiT method, the CNN based VN model and single-channel based deep learning method (ZF-GAN), the proposed PIC-GAN framework outperformed them remarkably at different acceleration factors showing the effectiveness of our method.

**Figure 7:**
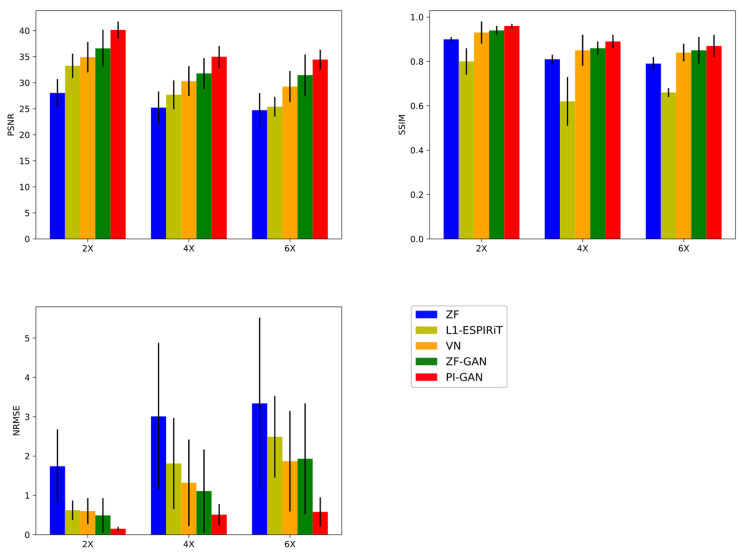
Performance comparisons (PSNR, SSIM and NMSE $\times \;10^{-5}$) on abdominal MRI data with different acceleration factors. (GT: Ground truth. ZF: Zero-filled. L1-ESPIRiT: Sparsity-based parallel imaging. VN: Variational network. ZF-GAN: Conventional GAN with single-channel images as input PIC-GAN: Our proposed method.)

As shown in Figure 7, the proposed PIC-GAN method significantly outperformed the L1-ESPIRiT, VN and ZF-GAN reconstruction with acceleration factors of 2, 4 and 6 with respect to all metrics (*p* < 0.01) for the abdominal data with regular Cartesian undersampling.

The reconstruction time of L1-ESPIRiT was calculated with 30 iterations of conjugate gradient descent using the BART toolbox. For the abdominal data, it took about 66 seconds, which was 165 times longer than the PIC-GAN based approaches. In contrast, ZF-GAN and PIC-GAN methods took about 0.4 to 0.7 seconds for the reconstruction of a single slice, which was much more time-efficient. Similarly, as shown in Table 2, the reconstruction time using PIC-GAN is much shorter than L1-ESPIRiT for the knee data, and comparable to other methods.

## 4. Discussion

In this study, we have developed a PIC-GAN model incoperating PI and GAN to improve the multi-channel MRI reconstruction. Experimental results show that our PIC-GAN outperformed conventional L1-ESPIRiT and the state-of-the-art VN and ZF-GAN methods in terms of all quantitative metrics. In addition, the speed of PIC-GAN reconstruction is faster than conventional L1-ESPIRiT, indicating its feasibility for real-time imaging.

Currently, several novel GAN-based approaches have been proposed for MRI reconstruction. For example, the DA-FWGAN [24] architecture used a fine-tuning method for training the neural network and the Wasserstein distance as the discrepancy measure between the reference and reconstructed images. SARA-GAN [26] integrated the self-attention mechanism with relative average discriminator to reconstruct images with more realistic details and better integrity. Meanwhile, in contrast to most supervised deep learning reconstruction method, an unsupervised GAN based approach [25] was proposed for accelerated imaging where fully-sampled datasets are difficult to be obtained. However, these approaches are limited to single-channel reconstruction, which is not suitable for modern MRI scanners. Besides, some artifacts removal techniques, e.g., motion correction [40], are also based on multi-channel acquisitions. Thus, single-channel reconstructions are less realistic for clinical routines since modern MRI scanners are equipped with multi-coils. Thus, several methods have been explored to address this problem. The variational network [27] approach was proposed to learn an end-to-end reconstruction procedure for complex-valued multi-channel imaging. Moreover, a similar result using a PI-CNN network was reported in [28] to integrate multi-channel *k*-space data and to exploit them through PI. However, the PI algorithm was not incorporated into the optimization equation of the network but only treated as a regularization term. In addition, Deepcomplex-CNN [29] was presented to directly map aliased multi-channel images to the reference images without the requirement of any prior information. Obviously, the data fidelity term of these approaches was only defined in a single-domain (either the image or frequency domain). In our proposed PIC-GAN, we used a progressive refinement method in both frequency and image domains, which can not only help to stabilize the optimization of the network, but also make full use of the complementary information of the two domains. More specifically, the loss function in the image domain ensures reducing aliasing artifacts between the reconstructed images and their corresponding ground truth (i.e., fully-sampled reconstructions). In addition, we want to emphasize that we separated the loss function of the *k*-space into two parts: one is used to guarantee that the reconstructed image generates its the corresponding undersampled image with matching undersampled *k*-space data, the other to minimize the discrepancies between the missing data and the data interpolated by PIC-GAN in the *k*-space. This ensures high-fidelity reconstructions with high acceleration factors.

It is crucial to mention that both ZF-GAN and PIC-GAN have outperformed the L1-ESPIRiT in terms of reconstruction robustness, speed and image quality. This is because the CS method is sensitive to the regularization term while deep learning-based approaches do not need to impose the sparsity assumption. The networks automatically learn the underlying features and aliasing artifacts of the reconstructed image. Thus, their performance is more robust compared to the conventional non-deep learning CS techniques. Furthermore, the CS method treats each reconstruction as an individual nonlinear optimization problem. In contrast, deep learning based methods pre-calculate the network parameters offline. Therefore, once the parameters of the PIC-GAN are determined, the reconstruction is super-fast to unseen data with the same undersampling factor since no iterative calculations are required. Besides, experimental results show the feasibility of our PIC-GAN to learn the mapping from undersampled artifact-corrupted images to the GT images, using different sampling patterns with fixed undersampling factor. This indicates that a fixed undersampling pattern is not a prerequisite to train the network.

Multi-channel imaging is widely used in current clinical practice. It is obvious that the multi-channel network achieves better performance than the combined single-channel reconstruction, demonstrating the multi-channel network has the advantage over single-channel reconstruction by incorporating the sensitivity maps within the network. The results suggest that the operation of introducing a sensitivity map during training is similar to applying a low-pass filter that not only discards high-frequency noise but also enables a fairly clear image to be reconstructed. However, as the acceleration factor increases, the input *k*-space starts to contain very few ACS lines, which results in a relatively poor quality of the generated sensitivity maps for training. Thus, possible extensions of PIC-GAN may be either to improve the accuracy of the sensitivity maps estimation or to incorporate a calibrationless [41,42] algorithm into the model.

This study has several limitations. First, system imperfections exist during data acquisition that were not considered in the current study. Further studies should be taken to include those physical imperfections, e.g., gradient delays, B0 inhomogeneity, multiple projections with opposing orientations, etc. Second, the sample size was relatively small and only included healthy subjects. Future investigations should enlarge the sample size and validate the model on patients to see its generalization performance. Third, a future study is warranted to evaluate the performance of the proposed PIC-GAN for higher acceleration rates. It is of note that although we have reported the average reconstruction time for our comparison study, the reconstruction efficiency also depends on the system configuration, e.g., actual GPU allocated etc.

## 5. Conclusions

In conclusion, by coupling multi-channel information and GAN, our PIC-GAN model has been successfully evaluated using two MRI datasets. The proposed PIC-GAN method not only demonstrated superior reconstruction efficacy and generalization capacity, but also outperformed conventional L1-ESPIRiT and other deep learning based algorithms with different acceleration factors. In terms of the reconstruction efficiency, our PIC-GAN can remarkably reduce the reconstruction time (from seconds to milliseconds per slice) for multi-channel data compared to iterative L1-ESPIRiT, which is promising for real-time imaging in a lot of clinical applications.

## Figures and Tables

**Table 1:** Performance comparisons (Normalized Mean Square Error (NMSE) $\times \;10^{-5}$, Structural Similarity Index (SSIM), Peak Signal to Noise Ratio (PSNR) and Average Reconstruction Time(s)) on abdominal magnetic resonance imaging (MRI) data with different acceleration factors. The bold numbers highlight the best results. The PIC-GAN outperformed the competing algorithms with significantly higher PSNR, SSIM and lower NMSE values (*p* < 0.05).

R	METHOD	REGULAR			RANDOM			TIME (s)
		PSNR	SSIM	NMSE	PSNR	SSIM	NMSE	
2-FOLD	ZF	28.03 ± 2.68	0.90 ± 0.01	1.74 ± 0.94	34.66 ± 2.98	0.95 ± 0.01	0.49 ± 0.33	**0.05 ± 0.01**
	L1-ESPIRiT	33.25 ± 2.34	0.8 ± 0.06	0.62 ± 0.25	33.69 ± 1.48	0.81 ± 0.03	0.50 ± 0.02	143.71 ± 1.20
	VN	34.99 ± 2.09	0.89 ± 0.03	0.51 ± 0.27	33.20 ± 2.82	0.90 ± 0.02	0.92 ± 0.63	0.38 ± 0.01
	ZF-GAN	34.91 ± 2.92	0.93 ± 0.05	0.60 ± 0.33	37.22 ± 1.77	0.96 ± 0.01	0.32 ± 0.09	0.37 ± 0.00
	PIC-GAN	**36.60 ± 3.57**	**0.94 ± 0.02**	**0.49 ± 0.44**	**39.59 ± 2.64**	**0.97 ± 0.01**	**0.19 ± 0.13**	0.69 ± 0.00
4-FOLD	ZF	25.21 ± 3.13	0.81 ± 0.02	3.01 ± 1.87	27.31 ± 3.23	0.84 ± 0.02	0.21 ± 0.15	**0.05 ± 0.01**
	L1-ESPIRiT	27.69 ± 2.79	0.62 ± 0.11	1.81 ± 1.16	27.87 ± 0.78	0.70 ± 0.03	1.54 ± 0.46	143.01 ± 1.13
	VN	30.30 ± 2.88	0.85 ± 0.07	1.32 ± 1.10	30.72 ± 2.31	0.87 ± 0.02	1.12 ± 0.51	0.38 ± 0.00
	ZF-GAN	31.79 ± 2.95	0.86 ± 0.03	1.11 ± 1.06	32.95 ± 2.57	0.89 ± 0.02	0.92 ± 0.64	0.36 ± 0.00
	PIC-GAN	**34.99 ± 2.09**	**0.89 ± 0.03**	**0.51 ± 0.27**	**33.20 ± 2.82**	**0.90 ± 0.02**	**0.92 ± 0.63**	0.69 ± 0.01
6-FOLD	ZF	24.71 ± 3.31	0.79 ± 0.03	3.34 ± 2.18	25.15 ± 3.37	0.79 ± 0.03	0.31 ± 0.21	**0.05 ± 0.01**
	L1-ESPIRiT	25.40 ± 1.88	0.66 ± 0.02	2.49 ± 1.04	25.71 ± 2.94	0.67 ± 0.01	2.49 ± 1.30	143.43 ± 2.18
	VN	29.26 ± 2.98	0.84 ± 0.04	1.87 ± 1.28	20.76 ± 2.64	0.84 ± 0.01	1.54 ± 0.97	0.39 ± 0.01
	ZF-GAN	31.45 ± 4.00	0.85 ± 0.06	1.93 ± 1.41	30.91 ± 2.72	0.85 ± 0.02	1.42 ± 1.01	0.40 ± 0.00
	PIC-GAN	**34.43 ± 1.92**	**0.87 ± 0.05**	**0.58 ± 0.37**	**31.76 ± 3.04**	**0.86 ± 0.02**	**1.22 ± 0.97**	0.68 ± 0.01

**Table 2:** Performance comparisons (NMSE $\times \;10^{-5}$, SSIM, PSNR and Average Reconstruction Time(s)) on knee MRI data with different acceleration factors. The bold numbers highlight the best results. The PIC-GAN outperformed the competing algorithms with significantly higher PSNR, SSIM and lower NMSE values (*p* < 0.05).

R	METHOD	REGULAR			RANDOM			TIME (s)
		PSNR	SSIM	NMSE	PSNR	SSIM	NMSE	
2-FOLD	ZF	25.95 ± 1.42	0.83 ± 0.03	5.25 ± 1.21	25.94 ± 1.19	0.83 ± 0.01	5.28 ± 1.13	**0.02 ± 0.01**
	L1-ESPIRiT	31.60 ± 1.27	0.72 ± 0.01	0.89 ± 0.55	30.07 ± 1.00	0.73 ± 0.02	1.01 ± 0.61	67.18 ± 1.10
	VN	32.79 ± 1.42	0.85 ± 0.02	0.60 ± 0.12	32.54 ± 1.43	0.86 ± 0.01	0.57 ± 0.12	0.19 ± 0.01
	ZF-GAN	34.71 ± 1.31	0.86 ± 0.00	0.44 ± 0.08	34.45 ± 1.60	0.87 ± 0.00	0.39 ± 0.10	0.22 ± 0.01
	PIC-GAN	**37.80 ± 1.02**	**0.91 ± 0.00**	**0.33 ± 0.09**	**37.98 ± 1.02**	**0.91 ± 0.00**	**0.10 ± 0.02**	0.43 ± 0.01
4-FOLD	ZF	24.27 ± 1.41	0.78 ± 0.03	8.05 ± 1.89	24.21 ± 1.23	0.78 ± 0.02	8.04 ± 1.89	**0.02 ± 0.00**
	L1-ESPIRiT	30.67 ± 1.38	0.59 ± 0.07	1.12 ± 0.57	28.98 ± 1.27	0.60 ± 0.01	1.27 ± 0.22	66.12 ± 1.13
	VN	31.65 ± 1.31	0.84 ± 0.02	0.82 ± 0.21	31.23 ± 1.26	0.83 ± 0.01	0.92 ± 0.20	0.19 ± 0.01
	ZF-GAN	33.28 ± 1.27	0.85 ± 0.01	0.69 ± 0.19	33.10 ± 1.26	0.84 ± 0.01	0.73 ± 0.17	0.21 ± 0.01
	PIC-GAN	**36.49 ± 1.30**	**0.89 ± 0.01**	**0.46 ± 0.15**	**36.17 ± 0.94**	**0.88 ± 0.01**	**0.58 ± 0.12**	0.44 ± 0.01
6-FOLD	ZF	23.18 ± 1.45	0.75 ± 0.04	8.09 ± 1.91	22.44 ± 1.46	0.76 ± 0.04	8.98 ± 2.31	**0.02 ± 0.00**
	L1-ESPIRiT	28.01 ± 0.98	0.55 ± 0.00	1.28 ± 0.24	27.52 ± 1.09	0.57 ± 0.01	1.59 ± 0.10	66.02 ± 1.76
	VN	30.01 ± 1.01	0.81 ± 0.01	1.18 ± 0.31	28.54 ± 1.22	0.80 ± 0.00	0.98 ± 0.10	0.20 ± 0.01
	ZF-GAN	31.47 ± 1.05	0.82 ± 0.01	0.93 ± 0.29	30.48 ± 1.24	0.81 ± 0.01	0.86 ± 0.11	0.24 ± 0.01
	PIC-GAN	**34.10 ± 1.09**	**0.86 ± 0.01**	**0.80 ± 0.26**	**33.85 ± 1.11**	**0.85 ± 0.00**	**0.81 ± 0.10**	0.45 ± 0.01

## Data Availability

Publicly available datasets were analyzed in this study. This data can be found here: http://old.mridata.org/undersampled/abdomens and http://mridata.org/fullysampled/knees (accessed on 18 October 2020).
